# Arabidopsis Myrosinase Genes *AtTGG4* and *AtTGG5* Are Root-Tip Specific and Contribute to Auxin Biosynthesis and Root-Growth Regulation

**DOI:** 10.3390/ijms17060892

**Published:** 2016-06-07

**Authors:** Lili Fu, Meng Wang, Bingying Han, Deguan Tan, Xuepiao Sun, Jiaming Zhang

**Affiliations:** Institute of Tropical Bioscience and Biotechnology, Key Laboratory of Tropical Crops Biology and Genetic Resources, Ministry of Agriculture; Hainan Bioenergy Center, Chinese Academy of Tropical Agricultural Sciences, Xueyuan Road 4, Haikou 571101, China; fulili@itbb.org.cn (L.F.); wangmeng1981_wm@126.com (M.W.); hanbingying@itbb.org.cn (B.H.); tandeguan@itbb.org.cn (D.T.); sunxuepiao@itbb.org.cn (X.S.)

**Keywords:** myrosinase, root-tip, *AtTGG4*, *AtTGG5*, DR5, flood tolerance, double mutant

## Abstract

Plant myrosinases (β-thioglucoside glucohydrolases) are classified into two subclasses, Myr I and Myr II. The biological function of Myr I has been characterized as a major biochemical defense against insect pests and pathogens in cruciferous plants. However, the biological function of Myr II remains obscure. We studied the function of two Myr II member genes *AtTGG4* and *AtTGG5 in Arabidopsis*. RT-PCR showed that both genes were specifically expressed in roots. GUS-assay revealed that both genes were expressed in the root-tip but with difference: *AtTGG4* was expressed in the elongation zone of the root-tip, while *AtTGG5* was expressed in the whole root-tip. Moreover, myrosin cells that produce and store the Myr I myrosinases in aboveground organs were not observed in roots, and *AtTGG4* and *AtTGG5* were expressed in all cells of the specific region. A homozygous double mutant line *tgg4tgg5* was obtained through cross-pollination between two T-DNA insertion lines, *tgg4E8* and *tgg5E12*, by PCR-screening in the F2 and F3 generations. Analysis of myrosinase activity in roots of mutants revealed that *AtTGG4* and *AtTGG5* had additive effects and contributed 35% and 65% myrosinase activity in roots of the wild type Col-0, respectively, and myrosinase activity in *tgg4tgg5* was severely repressed. When grown in Murashiege & Skoog (MS) medium or in soil with sufficient water, Col-0 had the shortest roots, and *tgg4tgg5* had the longest roots, while *tgg4E8* and *tgg5E12* had intermediate root lengths. In contrast, when grown in soil with excessive water, Col-0 had the longest roots, and *tgg4tgg5* had the shortest roots. These results suggested that *AtTGG4* and *AtTGG5* regulated root growth and had a role in flood tolerance. The auxin-indicator gene *DR5::GUS* was then introduced into *tgg4tgg5* by cross-pollination. *DR5::GUS* expression patterns in seedlings of F1, F2, and F3 generations indicated that *AtTGG4* and *AtTGG5* contributed to auxin biosynthesis in roots. The proposed mechanism is that indolic glucosinolate is transported to the root-tip and converted to indole-3-acetonitrile (IAN) in the tryptophan-dependent pathways by AtTGG4 and AtTGG5, and IAN is finally converted to indole-3-acetic acid (IAA) by nitrilases in the root-tip. This mechanism guarantees the biosynthesis of IAA in correct cells of the root-tip and, thus, a correct auxin gradient is formed for healthy development of roots.

## 1. Introduction

Glucosinolates are a group of S-linked secondary metabolites, occurring in the order Capparales, including the cruciferous crops and the model plant *Arabidopsis thaliana* [1,2,3]. These compounds are derived from amino acids and modified amino acids and, thus, more than 140 glucosinolate structures have been identified [4]. In *A. thaliana*, at least 37 glucosinolates with side chains derived from methionine, tryptophan, phenylalanine, and leucine were identified [5]. The glucosinolate profile in a certain cruciferous plant may also change at qualitative and quantitative levels during plant development and/or upon biotic and abiotic stresses, through regulated biosynthesis, and breakdown of glucosinolates [6,7,8,9].

Glucosinolates are hydrolyzed by a class of myrosinases (β-thioglucoside glucohydrolases, TGG, EC 3.2.1.147) into toxic compounds, such as nitriles, isothiocyanates, epithionitriles, and thiocyanates. Therefore, the glucosinolate-myrosinase system serves as a major chemical defense mechanism against pest insects, and bacterial and fungal pathogens [1,10]. Myrosinases are composed of two subfamilies, Myr I and Myr II [11]. Myr I activity has been detected in all of the glucosinolate-containing plants and tissues that have been investigated, and the coding genes have been cloned from *Sinapis alba* [12], *Brassica napus* [13], and *A. thaliana* [14,15]. All crucifers analyzed so far have multiple forms of Myr I. In oilseed rape (*B. napus*), about 25–30 myrosinase isoforms may be present [1]. These myrosinases were classified to MA, MB, and MC subfamilies, and were later grouped into a single Myr I subclass when the new type of myrosinases Myr II were identified [11,16]. The Myr I myrosinases are usually localized in specialized myrosin cells in all tissues of *Brassica* species and *A. thaliana* [17,18,19,20], while the substrate glucosinolates are localized in the “aleurone-like” cells in the seedlings [21] and/or “S-cells” in flower stalk [22]. Myrosinases and glucosinolates are mixed upon tissue disruption by pest insects and pathogens, thus providing chemical defense.

*AtTGG4* and *AtTGG5* were the first found Myr II genes [23,24], and their myrosinase activities of the recombinant proteins were confirmed by over-expressing the genes in *Pichia* [24]. Another member *AtTGG6* in *A. thaliana* was previously considered as a pseudogene due to several frame-shift mutations [25], but it was specifically expressed in anthers, similar to *AtTGG3*, another myrosinase pseudogene in *A. thaliana* [26]. However, functional alleles of *AtTGG6* were identified in some *A. thaliana* ecotypes recently [27].

Myr II subfamily members are distinct from Myr I subfamily members not only by sequence divergence, but also by gene structure and unusual intron utilization. To our knowledge, all Myr I subfamily genes have 12 exons, while Myr II have 13 exons. Unusual intron splice boundaries are present in myrosinase genes [28]. All known Myr I myrosinases use the GC..AG intron splice border for intron 1. However, the Myr II member genes *AtTGG4*, *AtTGG5*, and *AtTGG6* in *A. thaliana* and *AlTGG4*, *AlTGG5*, and *AlTGG6* in *Arabidopsis lyrata* use the GC..AG splice border for intron 10 instead [11,27], suggesting a different evolutionary scenario of Myr I and Myr II genes. In addition, *AtTGG5* has a second GC..AG intron splice border for intron 3. It is unknown why myrosinase genes use rare intron splicing at such a high frequency. Two Myr II member genes *CpTGG1* and *CpTGG2* were cloned from *Carica papaya* [11,16]. These two myrosinase genes contained conserved Myr II gene structure, however, they did not contain any unusual intron splicing border, supporting the hypothesis that *CpTGG1* and *CpTGG2* were the primitive form of myrosinase genes, and Myr II subfamily may represent the ancestor of myrosinase family [11,16]. Myrosinase was suggested to be evolved from cyanogenic *O*-β-glucosidase [1]. However, none of the characterized genes of cyanogenic *O*-β-glucosidase contain an unusual intron splice border, and papaya is the rare plant species that contain both glucosinolates and cyanogenic *O*-β-glucosides [29,30,31].

The biological function of Myr I myrosinases has been extensively studied, however, the function of Myr II members remain poorly understood. In this study, we report the involvement of two Myr II members, *AtTGG4* and *AtTGG5*, in auxin biosynthesis and root growth regulation.

## 2. Results

### 2.1. Root-Tip Specific Expression of AtTGG4 and AtTGG5

**Figure 1:**
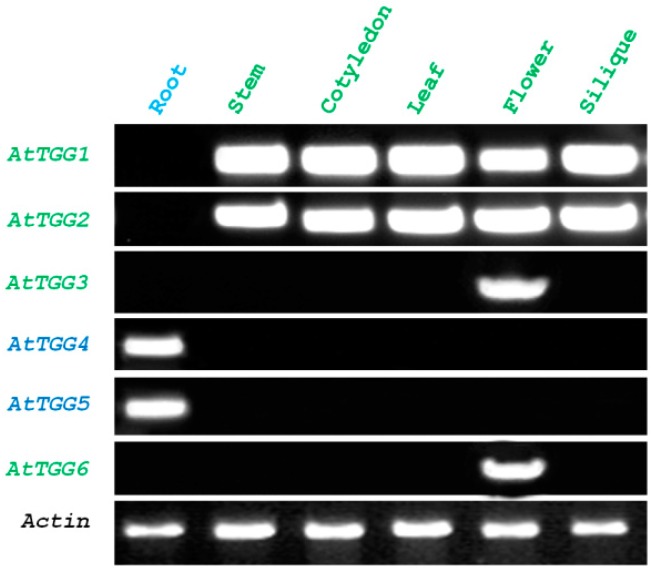
RT-PCR analysis of the myrosinase gene family in *Arabidopsis thaliana*. An actin gene (*ACT2*, U41998) was used as control.

RT-PCR analysis of the myrosinase gene family in *A. thaliana* revealed root-specific expression of *AtTGG4* and *AtTGG5*, while other myrosinase genes were not transcribed in roots (Figure 1). *AtTGG1* and *AtTGG2* were expressed in all aboveground organs, including stem, leaf, cotyledon, flower, and silique, whereas *AtTGG3* and *AtTGG6* were only expressed in the flower (Figure 1), suggesting functional allocations of the myrosinase gene family.

**Figure 2:**
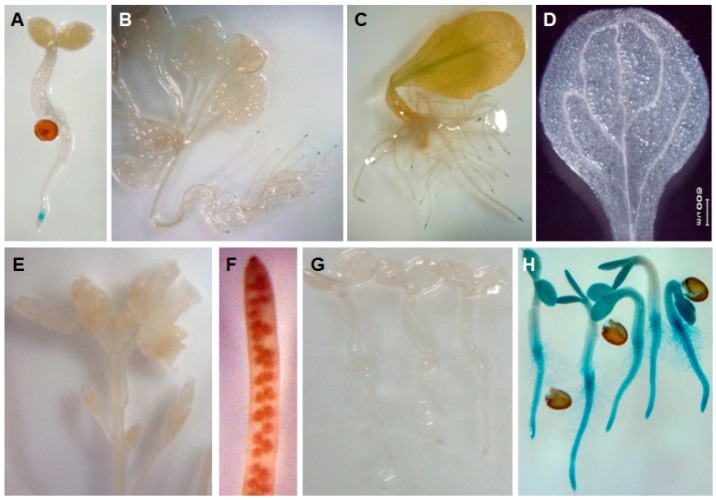
Gene expression pattern of *AtTGG4 Prom::GUS*. (**A**) A four-day-old seedling; (**B**) a two-week-old plant; (**C**) roots induced from the petiole of a leaf showing root-tip specific GUS staining; (**D**) a leaf; (**E**) an inflorescence; (**F**) a silique; (**G**) wild-type (negative control); and (**H**) transgenic plants with *CaMV 35S::GUS* (positive control).

To further characterize the expression pattern of *AtTGG4* and *AtTGG5*, their promoters were fused with *GUS* gene and transformed into *A. thaliana* Col-0. GUS staining revealed that *AtTGG4* was expressed at the elongation zone of the primary root-tips (Figure 2A) and the lateral root-tips (Figure 2B). The regenerated roots induced from leaf petioles of the transgenic plants also showed root-tip specific expression (Figure 2C). The aboveground organs, including cotyledon, leaf, flower stalk, flower, silique, and immature embryos were not observed to have positive GUS staining (Figure 2D–F). The wild-type Col-0 did not exhibit any GUS staining (Figure 2G), while the positive control transformed with *CaMV 35S::GUS* showed constitutional expression (Figure 2H).

**Figure 3:**
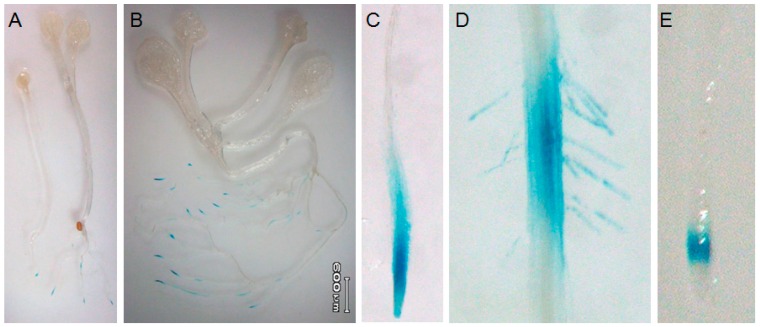
Gene expression pattern of *AtTGG5 Prom::GUS*. (**A**) Four-day-old seedlings; (**B**) a two-week-old plants; (**C**) a zoomed-in image of a root-tip showing the expression region of *AtTGG5 Prom::GUS* in the whole tip; (**D**) a zoomed-in image of a hairy zone; and (**E**) a zoomed-in image of a root-tip of *AtTGG4 Prom::GUS* presented here for easier comparison between *AtTGG4* and *AtTGG5*.

*AtTGG5 Prom::GUS* was primarily expressed at root-tips of primary and lateral roots with an expressing dense center at the elongation zone (Figure 3A,B). Its expression was not detected in aboveground organs. In contrast to *AtTGG4*, *AtTGG5* was expressed at the whole root-tip including the root cap, the division zone, the elongation zone, and the zone of differentiation (Figure 3C), and also in some of the hairy zones (Figure 3D), while the expression of *AtTGG4 Prom::GUS* was limited to the elongation zone of the root-tips (Figure 3E). Therefore, *AtTGG5* had a larger expression region and a higher expression level in roots, and had possibly more important biological function.

### 2.2. Screening of Homozygous Double T-DNA Insertion Mutants and Myrosinase Activity Test

**Figure 4:**
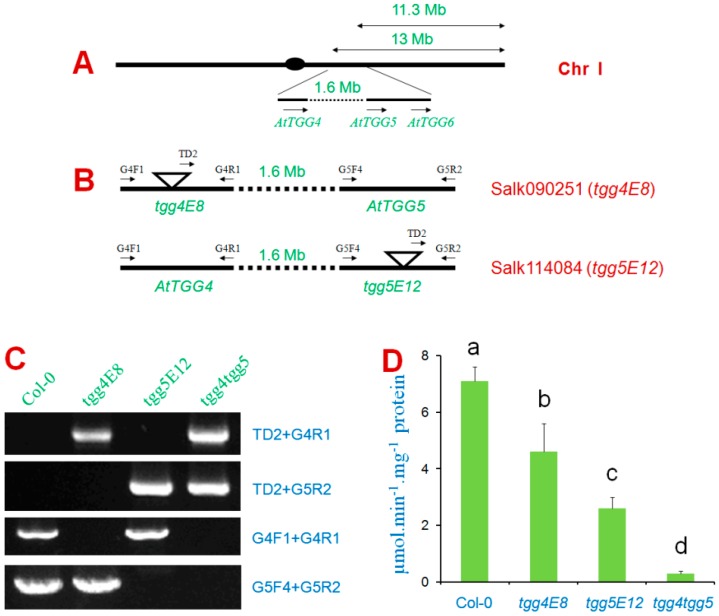
Schematic presentations of PCR primers and verification of single and double gene mutants in *A. thaliana*. (**A**) Relative locations of three Myr II genes in Chromosome I (Chr I) of *A. thaliana*; (**B**) schematic of PCR primers used to identify T-DNA insertion and homozygous mutants; the triangles stand for the T-DNA insertions in the relevant genes; (**C**) confirmation of T-DNA insertion in the single and double mutants using four primer pairs; and (**D**) myrosinase activity in roots of the single and double mutants; different letters on the columns indicate significant difference at 1% significant level.

Knock-out mutations are widely used to study the biological functions of genes. *AtTGG4* and *AtTGG5* are closely linked in Chromosome I of *A. thaliana* with a distance of 1.6 Mb (Figure 4A). To study the biological function of *AtTGG4* and *AtTGG5*, two T-DNA insertion lines were obtained from the Arabidopsis Biological Resource Center (ABRC). The flanking regions of the T-DNA were PCR amplified with a T-DNA specific primer and gene specific primers as shown in Figure 4B. The fragments were sequenced to determine the accurate position of T-DNA insertion. Mutant line Salk090251 contained a T-DNA insertion at +1650 (from start codon) in exon 8 of *AtTGG4* and, thus, designated as *tgg4E8* hereafter. Mutant line Salk114084 contained a T-DNA insertion at +2455 (from start codon) in exon 12 of *AtTGG5*, thus designated as *tgg5E12* hereafter.

In total, four primer pairs were used to determine whether a mutant plant possessed a homozygous T-DNA insertion (Figure 4C). The T-DNA specific primer TD2 and a gene specific primer were used to determine the existence of T-DNA insertion, and two gene specific primers located at 5′ and 3′ ends were used to amplify the allele without the T-DNA insertion. Thus, the homozygous *tgg4E8* was verified by PCR negative with primer pairs G4F1 + G4R1 and TD2 + G5R2, and PCR positive with primer pairs G5F4 + G5R2 and TD2 + G4R1 (Figure 4C) and, accordingly, the homozygous *tgg5E12* was verified *vice versa* (Figure 4C).

The homozygous *tgg4E8* and *tgg5E12* were cross-pollinated, and plants of the F2 generation were screened for recombinant events between the *AtTGG4* and *AtTGG5* loci using the PCR method. Seven out of 167 F2 plants were confirmed to have the desired linkage pattern of *tgg4E8-tgg5E12*. These plants were self-pollinated and seven F3 populations were generated. Ninety-seven F3 offspring were screened, and 27 homozygous double-mutant *tgg4tgg5* were obtained, accounting for 27.8% of the offspring, no less than 25% (the theoretic ratio), indicating that the double mutants did not have survivor problems in the greenhouse conditions.

Analysis of myrosinase activities in roots revealed that *tgg4E8* and *tgg5E12* possessed, respectively, 65% and 35% myrosinase activity of Col-0 (Figure 4D), suggesting that *AtTGG5* contributed most myrosinase activity in roots. The results were consistent with the GUS-staining results (Figure 3). Myrosinase activity in roots of the homozygous double mutant *tgg4tgg5* was very weak and almost undetectable (Figure 4D). In contrast, the aboveground organs of the single and double mutants and the wild-type possessed high myrosinase activity, approximately 20-fold as in the root of Col-0, without significant difference between genotypes (data not shown).

### 2.3. AtTGG4 and AtTGG5 Regulate Root Growth

**Figure 5:**
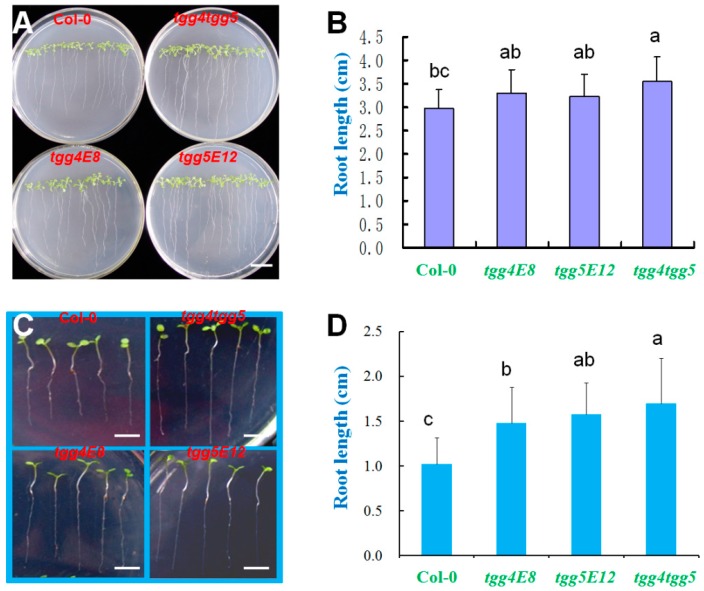
Root growth of *AtTGG4* and *AtTGG5* mutants on Murashiege & Skoog (MS) medium (**A**,**B**) and in soil with sufficient water (**C**,**D**). (**A**) Representative plates of four genotypes grown on MS medium for two weeks; scale bar represents 1 cm; (**B**) analysis of root lengths grown on MS medium; different letters above the columns indicate significant difference at 5% significant level; (**C**) representative seedlings of four genotypes grown in soil for two weeks after sowing; scale bars represent 5 mm; and (**D**) analysis of root lengths grown in soil; different letters indicate significant difference at 5% significant level.

To investigate the effects of *AtTGG4* and *AtTGG5* on root growth, the single and double mutants were grown on Murashieg & Skoog (MS) medium using Col-0 as control. Col-0 had the shortest roots among the four genotypes with an average length of 2.97 cm after two weeks of culture, while *tgg4tgg5* had the longest roots with an average length of 3.54 cm (Figure 5A), which were significantly longer than that of Col-0 (*p* ≤ 0.05, Figure 5B). The single mutants, *tgg4E8* and *tgg5E12*, both had slightly longer roots than Col-0, although it was not statistically significant (*p* > 0.05, Figure 5B). Similar results were observed in seedlings germinated in soil with sufficient water (Figure 5C,D), and Col-0 had the shortest roots (1.03 ± 0.23 cm), while *tgg4tgg5* had the longest roots (1.67 ± 0.42 cm) after sowing in soil for two weeks. These results suggested that *AtTGG4* and *AtTGG5* had a role in root-growth regulation, and they were possibly involved in auxin biosynthesis.

### 2.4. AtTGG4 and AtTGG5 Contribute to Auxin Biosynthesis in Roots

**Figure 6:**
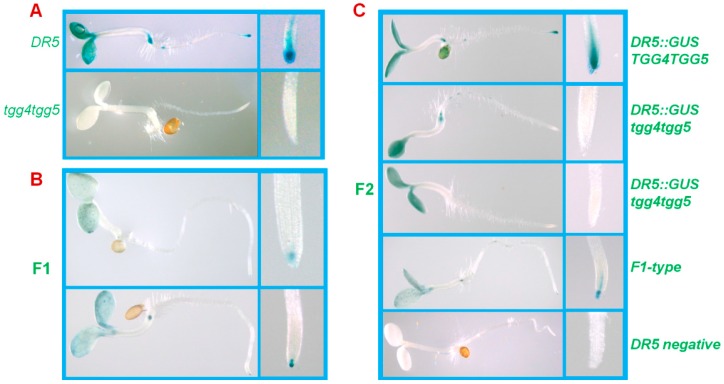
The role of *AtTGG4* and *AtTGG5* in auxin biosynthesis. (**A**) GUS staining of the parents: *DR5::GUS* and *tgg4tgg5*; (**B**) representative F1 plants; and (**C**) representative plants of the segregating F2 generation. Zoom-in images of the root-tips were presented beside each seedling.

To test the involvement of *AtTGG4* and *AtTGG5* in auxin biosynthesis, the well characterized *DR5::GUS* gene was used as an auxin indicator [32]. A DR5 line containing the *DR5::GUS* gene was cross-pollinated with *tgg4tgg5*. GUS-staining results revealed that *DR5::GUS* was primarily expressed in cotyledons, root-tips, the hypocotyl-root junctions of the DR5 parent line, and the expression in root-tips were centered at the cap region (Figure 6A). The *DR5::GUS* expression level in the F1 generation was lowered down in both cotyledons and root-tips due to the half dosage of *DR5::GUS*, *AtTGG4*, and *AtTGG5* genes (Figure 6B). In the F2 segregating generation, expression patterns similar to parents and F1 were identified. The plants that showed similar expression levels to DR5 in cotyledons, but lower or undetectable *GUS* expression in root-tips were also identified (Figure 6C), the genotype of these plants were *DR5::GUS*/*tgg4tgg5* (Figure 6C). Therefore, we concluded that *AtTGG4* and *AtTGG5* played a major role in auxin biosynthesis in root-tips.

### 2.5. AtTGG4 and AtTGG5 Confer Flood-Stress Tolerance in Arabidopsis

**Figure 7:**
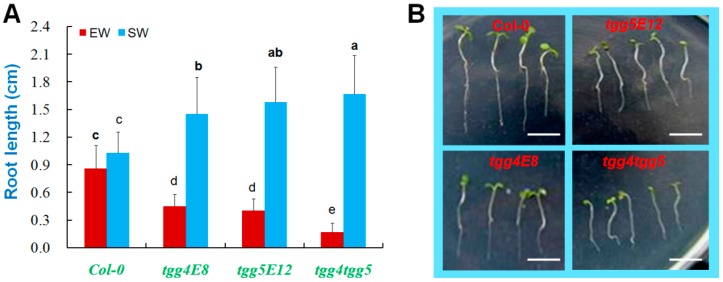
Root growth of *AtTGG4* and *AtTGG5* mutants in soil with excessive water. (**A**) Analysis of root length grown in soil with excessive water (EW) and sufficient water (SW) for two weeks after sowing; different letters above columns indicate significance at 5% significant level; and (**B**) representative seedlings of the four genotypes grown in soil with excessive water for two weeks after sowing; scale bars represent 5 mm.

To investigate the biological merits of *AtTGG4* and *AtTGG5*, the seeds of mutant lines were sown in soil with excessive water. The germination and growth of Col-0 was slightly affected with an average root length of 0.86 cm in two weeks after sowing (Figure 7A), but the mutants were seriously affected with water-logged hypocotyls and much shorter roots (Figure 7B), especially for *tgg4tgg5*. The average root length of *tgg4tgg5* was only 0.17 cm in length after two weeks of incubation, which was only 10% of the root length of Col-0, and some of the seedlings had no roots at all (Figure 7B). These results suggested that *AtTGG4* and *AtTGG5* genes were important to the root development of young seedlings in flooded conditions.

## 3. Discussion

### 3.1. Root-Tip Specific Expression Implicates a Role of AtTGG4 and AtTGG5 in Root Growth Regulation

AtTGG4 and AtTGG5 are the first myrosinases discovered in the MYR II myrosinase subfamily [11,23,24]. Other MYR II subfamily members: CpTGG1, CpTGG2, and AlTGG4–6 were then identified, respectively, in papaya and *Arabidopsis lyrata* [11,16,27]. The previously-reported inactive member gene *Attgg6* in *A. thaliana* [25], was recently reported to have functional alleles that were predominantly expressed in pollen grains and served as defense against insect herbivores [27].

*AtTGG4* and *AtTGG5* have been found to be root specific long ago [23], and their recombinant proteins over-expressed in *Pichia pastoris* had different catalytic properties compared to the MYR I myrosinases AtTGG1 and AtTGG2 [24]. However, their root-tip-specific expression and biological function in plants was unknown. Analysis of the expression pattern of a gene has been widely used as an important method to deduce its biological function. We firstly studied the expression pattern of the myrosinase gene family in *A. thaliana* by RT-PCR, and confirmed the results in previous reports [23,25,33] (Figure 1). To further characterize the expression pattern of *AtTGG4* and *AtTGG5*, we fused their promoters with the *GUS* reporter gene and transformed Col-0. GUS staining revealed that *AtTGG4* and *AtTGG5* were all predominantly expressed in root-tips with differences. *AtTGG4* was only expressed in the elongation zone of all types of roots (Figure 2), while *AtTGG5* was expressed in the whole root-tip, including the cap zone, the division zone, the elongation zone, and some hairy zones (Figure 3). However, the most densely expressed region for *AtTGG5* was still the elongation zone. It was the first time the root-tip specific expression for *AtTGG4* and *AtTGG5* and their involvement in the regulation of root growth was reported.

Enzymatic analysis of the mutants revealed that *AtTGG5* contributed 65% myrosinase activity in roots, while *AtTGG4* contributed 35% (Figure 4B), which was in agreement with the expression pattern of the two genes. Therefore, *AtTGG5* had a larger expression region and a higher expression level, and may have more important biological function compared to *AtTGG4*.

### 3.2. Screening of Homozygous Double T-DNA Insertion Mutants

*AtTGG4* and *AtTGG5* were located on the same arm of Chromosome I, with a distance of approximately 1.6 Mb (Figure 4A). To obtain a double mutant, two single T-DNA insertion mutants *tgg4E8* and *tgg5E12* carrying the same kanamycin resistant gene in T-DNA, were cross-pollinated, and the F2 population was screened for recombinant events between the *AtTGG4* and *AtTGG5* loci using the designated PCR protocol. We identified the desired linkage pattern of *tgg4E8*-*tgg5E12* at a rate of 4.2%, which was approximately half of the expected rate calculated according to the 1.6 Mb distance between the two genes [34]. The lower identification rate may be explained by the failure to identify some genotypes with the recombination events, for example, the genotype *tgg4E8-tgg5E12*/*TGG4-TGG5* would generate identical PCR patterns with *tgg4E8-TGG5*/*TGG4-tgg5E12* (Table 1). False positive PCR reactions may have also ruled out some desired individuals. Anyway, a total of seven plants with *tgg4E8*-*tgg5E12* linkage pattern were identified, and they were self-pollinated to generate F3 populations. Twenty-seven out of 97 F3 individuals were identified to be homozygous double mutants, accounting for 27.8% F3 plants. This ratio was close to the theoretical ratio, ignoring the cross-over between *AtTGG4* and *AtTGG5* loci, indicating that the double mutants did not have survivor problems in greenhouse conditions.

**Figure 8:**
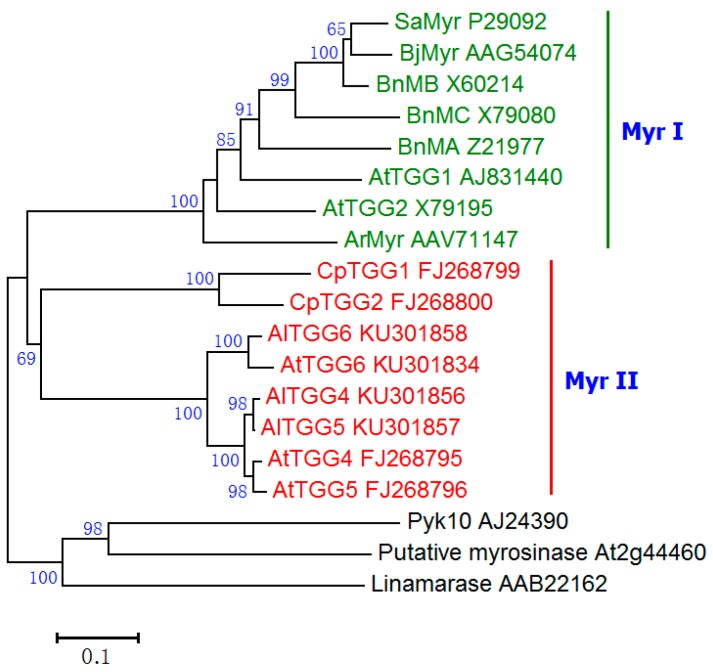
Evolutionary relationships of myrosinases and *O*-β-glucosidases. The evolutionary history was inferred using the neighbor-joining method [39]. The percentage of replicate trees in which the associated taxa clustered together in the bootstrap test (1000 replicates) are shown next to the branches [40]. Evolutionary analyses were conducted in MEGA7 [41]. The scale bar indicates 0.1 residue substitutions.

The myrosinase activity of the homozygous double mutant *tgg4tgg5* was almost undetectable. However, we also detected weak myrosinase activity, which was possibly from the non-specific hydrolysis of sinigrin by *O*-β-glucosidases. Some *O*-β-glucosidases in the leaves of *A. thaliana* have been demonstrated to have weak myrosinase activity [35]; however, no *O*-β-glucosidases in roots of *A. thaliana* have been shown to have myrosinase activity. Two putative myrosinase genes (*At2g44460* and *At3g09260* (*Pyk10*)) were demonstrated to be expressed in roots [36,37]. However, there was no evidence to prove that they catalyzed the hydrolysis of thioglucosides. Phylogenetic analysis indicated that these two proteins were not clustered in either the Myr I or the Myr II subfamily of myrosinases, but clustered with the linamarase from cassava (Figure 8). Moreover, all plant myrosinases use a glutamine residue to replace the general acid/base glutamate of *O*-β-glucosidases [11,16,38]. However, the two putative myrosinases (*At2g44460* and *At3g09260*) do not contain this replacement. Therefore, they are not likely myrosinases, but they might possess weak non-specific myrosinase activity.

### 3.3. AtTGG4 and AtTGG5 Regulate Root Growth and Confer Flood Tolerance

We have demonstrated that *AtTGG4* and *AtTGG5* were involved in root growth. The wild-type Col-0 did not show better phenotypes than their disabled mutant lines either on MS medium or in soil with sufficient water (Figure 5). When germinated on MS medium, Col-0 had the shortest roots, and *tgg4tgg5* had the longest roots, while the single mutants had intermediate root lengths (Figure 5A,B). Similar differences were observed in seedlings germinated in soil with sufficient water (Figure 5C,D), in which Col-0 had the shortest roots, while *tgg4tgg5* had the longest roots.

However, when seeds were sown in soil with excessive water, the growth of Col-0 seedlings was only slightly affected and the root length was similar to those grown in soil with sufficient water (Figure 7); but root growth of the mutant lines was significantly affected, especially for *tgg4tgg5*. The average root length of *tgg4tgg5* was only 10% of the root length of Col-0 (Figure 7). The single mutant lines *tgg4E8* and *tgg5E12* had intermediate phenotypes, supporting the additive effects of the two genes. These results suggested that *AtTGG4* and *AtTGG5* genes were very important to the development of young seedlings in flooded conditions. Flooding causes premature senescence, which results in leaf chlorosis, necrosis, defoliation, cessation of growth, and reduced yield [42]. The mechanism of *AtTGG4* and *AtTGG5* in flood stress tolerance is unknown. However, the two genes may function through the biosynthesis of indole-3-acetic acid (IAA) from indolic glucosinolates and, thus, promote root growth in flooded soil. Indolic glucosinolates make up nearly half of the total glucosinolate composition in roots and late-stage rosette leaves [6].

### 3.4. AtTGG4 and AtTGG5 Contribute to Auxin Biosynthesis through a Tryptophan-Dependent Pathway

**Figure 9:**
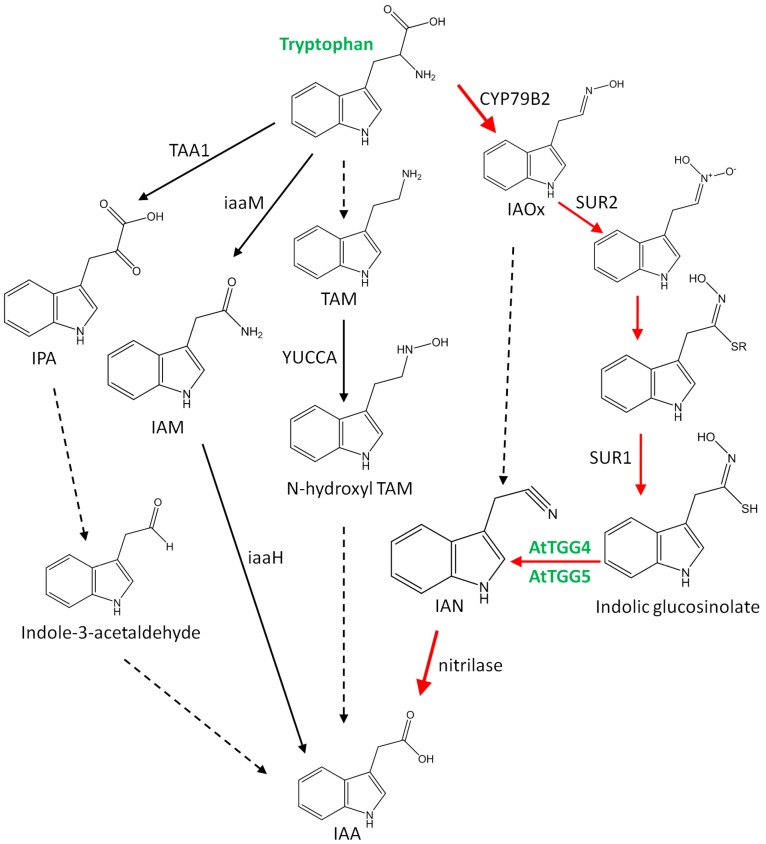
A model of the tryptophan-dependent auxin biosynthetic pathways. Modified from Zhao *et al.* [43]. The pathway that *AtTGG4* and *AtTGG5* are involved in is shown in red. Solid arrows indicate that the enzymes catalyzing the steps have been identified; Dashed arrows indicate proposed steps, in which enzymes catalyzing the steps have not been determined conclusively. TAM, tryptamine; IAOx, indole-3-acetaldoxime; IPA, indole-3-pyruvate; IAM, indole-3-acetamide; IAN, indole-3-acetonitrile; TGG, thioglucoside glucohydrolase (AtTGG4, AtTGG5).

We have proved the involvement of *AtTGG4* and *AtTGG5* in auxin biosynthesis by using the well-characterized auxin indicator, the *DR5::GUS* gene [32]. When the *DR5::GUS* gene was introduced into *tgg4tgg5* line by cross-pollination, the expression pattern of *DR5::GUS* gene was altered in many plants of the F2 generation, in which the root-tips had no or very low GUS staining compared to the parent DR5 line, while the cotyledons had as dense staining as the parent DR5 line (Figure 6). Therefore, we concluded that *AtTGG4* and *AtTGG5* played an important role in auxin biosynthesis in root-tips. Although most plants in the homozygous *DR5::GUS*/*tgg4tgg5* population possessed high *GUS* expression in cotyledons, but weak *GUS* expression in root-tips, a few plants that possessed the expression pattern similar to the parent DR5 line were observed. These results could be explained by the existence of other auxin biosynthetic pathways besides the AtTGG4 and AtTGG5 pathway in the root-tips (Figure 9).

Auxin biosynthesis in plants is extremely complicated [43]. Plants can synthesize auxin via many independent biosynthetic pathways, including at least four trytophan-dependent pathways and one tryptophan-independent pathway. Auxin can also be released from inactive conjugates by hydrolysis, such as IAA-methyl ester [44,45], IAA-amino acids, IAA-sugar, IAA-proteins, and peptides [46,47,48].

The tryptophan-dependent pathways have been extensively reviewed [43,48,49]. However, the involvement of myrosinases and glucosinolates in auxin biosynthesis is obscure. Glucosinolates are derived from amino acids [50,51], and cytochrome P450 has been known to play a key role in the conversion of amino acids to oxime in the biosynthesis of glucosinolates [52]. In the case of indolic glucosinolate, tryptophan was the precursor, and it was converted to indole-3-acetaldoxime (IAOx) by CYP79B2 in *A. thaliana* [53,54]. IAOx was then converted to indolic glucosinolate by SUR2 and SUR1, respectively [55,56]. Inactive mutation of SUR2 and SUR1 resulted in “high-auxin” phenotype and “super-root” [55,56]. Therefore, biosynthesis of indolic glucosinolate seemed to serve as a mechanism to reduce IAA level in roots [55,56]. However, our research indicated that the indolic glucosinolates may be transported to root-tips, and then hydrolyzed to indole-3-acetonitrile (IAN) by AtTGG4 and AtTGG5 (Figure 9). IAN is finally converted to IAA by nitrilases in the root-tips. Therefore, the biosynthesis of indolic glucosinolate may serve as a mechanism to guarantee the biosynthesis of IAA in correct cells in root-tip to form a proper auxin gradient for healthy development of roots. When this gradient was destroyed, as in *sur1* and *sur2* mutants, “super-root” occurred [55,56].

## 4. Materials and Methods

### 4.1. Plant Material and Growth Conditions

Arabidopsis ecotype Col-0 (N1092) was obtained from the Nottingham Arabidopsis Stock Centre (NASC), UK. The T-DNA insertion lines were obtained from the Arabidopsis Biological Resource Center (ABRC). The plants were grown at 20 °C, 16 h day photoperiod, and 200 μmol·m^−2^·s^−1^ light intensity. Roots were collected from two-week-old plants, cotyledons were collected from one-week-old plants, leaves were collected from 2–5 week old plants, and flowers and siliques were collected from 4–5 week old plants.

### 4.2. Expression Analysis of Myrosinase Gene Family by Reverse Transcription Polymerase Chain Reaction (RT-PCR)

Plant samples were ground in liquid nitrogen. Total RNA was isolated with 3S Trizol Total RNA isolation reagents (Shenergy Biocolor Bioscience and Technology Company, Shanghai, China). mRNA was then purified from the total RNA with an Oligotex Direct mRNA purification kit (Qiagen GmbH, Hilden, Germany). Moloney Murine Leukemia Virus (M-MuLV) reverse transcriptase (TaKaRa Biotechnologies, Dalian, China) was used to generate first strand cDNA. PCR was performed to study the spatial expression using gene specific primers. For *AtTGG4*, G4E1 (5′-ACCGTACGGGATTTCCCAG-3′) and G4E2 (5′-ATCCATCTCCATAGCCTTTGGT-3′) were used. For *AtTGG5*, G5E1 (5′-AACCGTAGTGGATTTCCCAA-3′) and G5E2 (5′-ATCCATTTCCATAGCCTTTGAG-3′) were used. For *AtTGG6*, G6F1 (5′-ACCCGCTGAAAAGCTCCATCAA-3′) and G6R1 (5′-GGCTTCCACTTATTTTGCAATGAACC-3′) were used [25]. For *AtTGG1*, G1E1 (5′-ATAAACCATGAAGCTTCTTATGC-3′) and G1E2 (5′-GATATTTCATGCATCTGCAAGA-3′) were used. For *AtTGG2*, G2E1 (5′-TAACCATGAAGCTTCTTGGGT-3′) and G2E2 (5′-GTTTTGGTCTTTCATGTGAGG-3′) were used. For *AtTGG3*, T7F4 (5′-GACGGCTATAGATTCTCGCTT-3′) and P12 (5′-ACGGCTATGTCGCAACA-3′) were used [26]. An actin gene (*ACT2*) was used as a control in the RT-PCR experiments with primers ActF1 (5′-GGCAAAAGGATGCTTATGTTGG-3′) and ActR1 (5′-ATTTCACGCTCTGCTGTGGTGG-3′) [57]. The PCR reactions were performed at 94 °C for 4 min, followed by 30 cycles of 94 °C for 30 s, 55 °C for 30 s, and 72 °C for 3 min.

### 4.3. Expression Analysis of AtTGG4 Prom::GUS and AtTGG5 Prom::GUS

The promoter of *AtTGG4* (1475 bp) was amplified from Col-0 with primers AP13 (5′-AGAGAAGCTTGTCGGTTTTGATTGGGTGAGAGA-3′, *Bam* HI site underlined) and AP14 (5′-AGTTGGATCCGGTTTGTATTTTCTTTATTGATGTGCTTC-3′, *Hind* III site underlined) and LA Taq DNA polymerase (TaKaRa Biotechnologies). *AtTGG5* promoter (1455 bp) was amplified with primers AP17 (5′-TGAGAAGCTTCCAGTTGGGTTTGGGTTAGTTTG-3′, *Bam* HI site underlined) and AP18 (5′-TGTTGGATCCGGTTTGTATTTTCTTTATTGATGGGCT-3′, *Hind* III site underlined). The products were digested with *Bam* HI and *Hind* III and ligated with the *Bam* HI and *Hind* III site of the binary vector *pBI121* to replace the cauliflower 35S promoter for the *GUS* gene. The constructs were transformed into *Agrobacterium tumefaciens* strain C58. Col-0 wild type plants were transformed using vacuum filtration [58]. Transformants were selected on medium containing 40 mg/L kanamycin. T2 homozygous transgenic lines were used for GUS staining according to a standard method [59].

### 4.4. Confirmation of T-DNA Insertion Mutants and Creation of Homozygous Double Mutant tgg4tgg5

The T-DNA insertion lines were grown as described above. DNA was isolated with a Plant Genomic DNA Isolation Kit (TaKaRa Biotechnologies). The T-DNA flanking sequences in *AtTGG4* mutant lines were amplified with a primer TD2 (5′-AACCCTATCTCGGGCTATTC-3′) located at the T-DNA border and an *AtTGG4* gene specific primer G4R1 (5′-CATATACAAAACACATAAGGTC-3′), and the T-DNA flanking sequences in the *AtTGG5* mutant lines were amplified with TD2 and an *AtTGG5* specific primer G5R2 (5′-AACACACAACAAGGTATAGGTA-3′). The locations and orientations of the primers were shown in Figure 8. The homozygous nature of the mutant lines was examined by amplification of *AtTGG4* and *AtTGG5* full length genomic DNA with primers pairs G4F1 (5′-ATCACCAAAAGAAGCACA-3′) and G4R1 for *AtTGG4*, and G5F4 (5′-TCATCACCAAAAGAAGCC-3′) and G5R2 for *AtTGG5*. The homozygous T-DNA insertion line would not be able to yield a PCR fragment for the relevant gene due to T-DNA insertion under designated PCR conditions (94 °C, 30 s; 60 °C, 30 s; 72 °C, 3 min, 35 cycles), while the wild type Col-0 would yield both *AtTGG4* and *AtTGG5* bands (Figure 4).

Although *AtTGG4* and *AtTGG5* loci were tightly linked with only a 1.6 Mb distance (Figure 4), the homozygous T-DNA insertion lines *tgg4E8* and *tgg5E12* were cross-pollinated to get homozygous double mutant lines *tgg4tgg5*. F1 individuals that were PCR positive with both primer pairs TD2 + G5R2 and TD2 + G4R1 were grown to get seeds. The proposed genotypes of the F1 gametes and F2 population were listed in Table 1. DNA was extracted from 157 F2 plants. Primer pairs TD2 + G4R1, TD2 + G5R2, G4F1 + G4R1, and G5F4 + G5R2 were used to screen for plants with the linkage pattern of *tgg4E8-tgg5E12*. The target plants were PCR positive with primer pairs TD2 + G4R1 and TD2 + G5R2, and PCR negative with either primer pair G4F1 + G4R1 or G5F4 + G5R2. The selected plants were self-pollinated to generate a F3 population, and screened with primer pairs TD2 + G4R1, TD2 + G5R2, G4F1 + G4R1, and G5F4 + G5R2 again. The homozygous double gene mutant should be PCR positive with both primer pairs TD2 + G4R1 and TD2 + G5R2, and PCR negative with both G4F1 + G4R1 and G5F4 + G5R2 (Figure 4C).

### 4.5. Analysis of Myrosinase Activity

Myrosinase activity in the roots of mutants and wild-type was measured, as described previously [11]. The glucose produced during enzymatic hydrolysis was measured by a glucose oxidase (GOD)-4-aminoantipyrine (PAP) test reagent (Shanghai Rongsheng Biotechnologies, Shanghai, China), as described previously [16].

### 4.6. Flood Tolerance Test

To test the influence of excessive water on seed germination and root growth, the seeds of mutants and wild-type were sown in sterilized soil and watered with sterilized tap water. For the normally watered control, excessive water was removed from the container that held the pots when the soil in the pots was completely wet. For flooding treatment, excessive water of approximately three fourths of the soil depth was always retained in the pots. Ten pots (five for flood treatment and five for control) were prepared for each genotype. The pots were placed in a clean growth chamber free of insects. The growth conditions were 16 h day photoperiod, 200 μmol·m^−2^·s^−1^ light intensity, and 20 °C. The root length of the seedlings was measured two weeks after sowing. The significance of the differences between genotypes was assayed by one-way ANOVA test at a 5% significance level, followed by LSD test.

### 4.7. The Role of AtTGG4 and AtTGG5 in Auxin Biosynthesis

To test the involvement of *AtTGG4* and *AtTGG5* in auxin biosynthesis, a DR5 line containing the *DR5::GUS* gene [32] was used as a pollen donor to cross-pollinate with the homozygous *tgg4tgg5* line. The F1 seedlings were stained as described above to verify the hybrid. F1 seedlings were grown under normal conditions as described above to get seeds for F2. A portion of F2 seedlings were stained to analyze the segregation of GUS expression patterns, while the rest of the F2 seedlings were grown in a growth chamber under normal conditions and were screened for homozygous *tgg4tgg5* genotypes with the PCR method as described above. The homozygous *tgg4tgg5* plants were grown to get seeds by self-pollination. The F3 seeds were germinated sterile and stained with GUS-reagent, and the F2 single plant lines with 100% GUS positive were regarded as homozygous *DR5::GUS*/*tgg4tgg5*.

## Figures and Tables

**Table 1:** Genotypes of gametes and F2 individuals of the hybrid *TGG4tgg5E12*/*tgg4E8TGG5*.

Gametes	TGG4-tgg5E12 (≈44%)	tgg4E8-TGG5 (≈44%)	TGG4-TGG5 (≈6%)	tgg4E8-tgg5E12 (≈6%)
TGG4-tgg5E12 (≈44%)	TGG4-tgg5E12	tgg4E8-TGG5	TGG4-TGG5	tgg4E8-tgg5E12
	TGG4-tgg5E12	TGG4-tgg5E12	TGG4-tgg5E12	TGG4-tgg5E12
	(≈19.4%)	(≈19.4%)	(≈2.6%)	(≈2.6%)
tgg4E8-TGG5 (≈44%)	TGG4-tgg5E12	tgg4E8-TGG5	TGG4-TGG5	tgg4E8-tgg5E12
	tgg4E8-TGG5	tgg4E8-TGG5	tgg4E8-TGG5	tgg4E8-TGG5
	(≈19.4%)	(≈19.4%)	(≈2.6%)	(≈2.6%)
TGG4-TGG5 (≈6%)	TGG4-tgg5E12	tgg4E8-TGG5	TGG4-TGG5	tgg4E8-tgg5E12
	TGG4-TGG5	TGG4-TGG5	TGG4-TGG5	TGG4-TGG5
	(≈2.6%)	(≈2.6%)	(≈0.36%)	(≈0.36%)
tgg4E8-tgg5E12 (≈6%)	TGG4-tgg5E12	tgg4E8-TGG5	TGG4-TGG5	tgg4E8-tgg5E12
	tgg4E8-tgg5E12	tgg4E8-tgg5E12	tgg4E8-tgg5E12	tgg4E8-tgg5E12
	(≈2.6%)	(≈2.6%)	(≈0.36%)	(≈0.36%)

The frequencies of genotypes in brackets were based on the assumption of an average 250 kb/cM for *A. thaliana* [34]; Recombinant gametes and F2 individuals with recombinant events were presented in grey.
